# The effect of grape products containing polyphenols on oxidative stress: a systematic review and meta-analysis of randomized clinical trials

**DOI:** 10.1186/s12937-021-00686-5

**Published:** 2021-03-12

**Authors:** Sahar Sarkhosh-Khorasani, Zohreh Sadat Sangsefidi, Mahdieh Hosseinzadeh

**Affiliations:** 1 Nutrition and Food Security Research Center, Shahid Sadoughi University of Medical Sciences, Yazd, Iran; 2 Department of Nutrition, School of Public Health, Shahid Sadoughi University of Medical Sciences, Yazd, Iran

**Keywords:** Grape, Polyphenols; oxidative stress; meta-analysis

## Abstract

**Background:**

The literature showed that Grape Products Containing Polyphenols (GPCP) had anti-oxidant activity. However, the effects of GPCP on different biomarkers of oxidative stress are still controversial. In this regard, this systematic review and meta-analysis aimed to evaluate the effect of Grape Products Containing Polyphenols (GPCP) intake on oxidative stress markers.

**Methods:**

PubMed, Scopus, Web of Science, and Google Scholar data bases were searched up to August 20, 2020. A random-effects model, weighted mean difference (WMD), and 95% confidence interval (CI) were applied for data analysis. Meta-analysis was conducted over 17 eligible RCTs with a total of 633 participants. The study registration number is CRD42019116696.

**Results:**

A significant increase was observed in Total Antioxidant Capacity (TAC) (weighted mean difference (WMD) = 1.524 mmol/L, 95% confidence interval (CI): 0.83, 2.21). Intake of GPCP enhanced Superoxide Dismutase (SOD) (WMD = 0.450 mmol/L, 95% CI: 0.23, 0.66), TAC (WMD = 2.829 mmol/L, 95% CI: 0.13, 5.52), and Oxygen Radical Absorbance Capacity (ORAC) (WMD = 0.524 μmol/L, 95% CI: 0.42, 0.62) among healthy participants. Higher GPCP doses increased SOD (WMD = 0.539 U/mgHb, 95% CI: 0.24, 0.82) and ORAC (WMD = 0.377 μmol/L, 95% CI: 0.08, 0.67), whereas longer intervention periods enhanced ORAC (WMD = 0.543 μmol/L, 95% CI: 0.43, 0.64).

**Conclusion:**

GPCP intake may partly improve status of oxidative stress, but further well-designed trials are required to confirm these results.

**Supplementary Information:**

The online version contains supplementary material available at 10.1186/s12937-021-00686-5.

## Introduction

Under normal physiological conditions, various enzymatic systems, such as superoxide dismutase (SOD), catalase, and *glutathione peroxidase* (*GPx*) act as antioxidants and protect the cells against free radical damage, including reactive oxygen species (ROS) [1, 2] Free radicals attack the main macromolecules and lead to cell damage and homeostasis. Increased number of free radicals reduce the detoxification capacity of tissues [3] and lead to oxidative stress. Oxidative stress occurs from the imbalance between production of ROS and protective effect of the antioxidant system, which is responsible for their neutralization and/or removal [4, 5]. Oxidative stress can be caused by biological endogenous factors [6, 7] or exogenous environmental factors [8]. High accumulation of free radicals overwhelms the antioxidant capacity of the body and leads to irreversible oxidative damage to nucleic acids, lipids, and proteins [9]. Oxidative damage lead to the cellular damage and cause changes in gene expression, cell proliferation, and apoptosis [9] Consequently, the aging process of the body is influenced and many chronic diseases develop, including cardiovascular disease, neural degeneration, cancer, and diabetes [2, 10]. Endogenous antioxidant defense system involves a network of antioxidant enzymes and non-enzymatic molecules in cytoplasm of organs [2]. Antioxidant enzymes, such as SOD, catalase, and glutathione reductase, transform ROS into more stable molecules and maintain oxidative equilibrium [2]. In addition, reduced glutathione (GSH) is a tripeptide made of glutamine, cysteine, and glycine with protective function against oxidative stress [4]. Malondialdehyde (MDA), as a byproduct of polyunsaturated fatty acids peroxidation [11], can be toxic, potentially mutagenic, and atherogenic due to its reaction with biomolecules such as protein and nucleic acid [12]. It is also a biomarker of oxidative stress [13]. Oxygen radical absorbance capacity (ORAC) has been widely used for measuring the antioxidant activity [14]. Considering the difficulty of measuring plasma antioxidant capacity of each sample and regarding the interactions among different compounds [15], the total antioxidant capacity (TAC) was evaluated [16]. Generally, increased oxidative stress decreases TAC [17].

Human trials showed protective effects of grape products containing polyphenols (GPCP) in disease which oxidative stress involved in them such as cardiovascular disease [18–22], type 2 diabetes [23, 24], metabolic syndrome components [25], dyslipidemia [26], neuro-degenerative [27], and some in-vitro studies investigating several cancers [28–30]. For instance, GPCP had anti-inflammatory effects in subjects with stable coronary artery disease [18] and diabetic patients [23], and as well as improving effects on insulin resistance and glycemic control in type 2 diabetic patients [24]. Moreover, GPCP decreased plasma lipids and oxidative stress in women [22] and markers of the metabolic syndrome in obese patients [25].The GPCP contain antioxidants in the form of polyphenols including phenolic acid (e.g. gallic acid), resveratrol, proanthocyanidin, and flavonoids (anthocyanins, flavonols, and quercetin) [31, 32]. These phenols are mostly present in grape crust, stems, leaves, and kernels in comparison with the juicy parts [33–35]. However, the results of randomized clinical trials (RCTs) over the effect of GPCP on oxidative stress markers are inconsistent.

Intake of 2 g/d grape polyphenol in overweight and obese participants with type 2 diabetes [24] also 0.5 g/d resveratrol (~ 500 mg polyphenol) supplementation [36] had no significant effect on antioxidant capacity. In addition, consuming 480 g/d grape juice (~ 945 mg polyphenol) [37] and 92 g/d grape powder consumption (~ 62.24 mg polyphenol) had no significant effect on ORAC levels [38]. While 90 g/d raisin (~ 178.75 mg polyphenol) increased ORAC levels [39]. Moreover, some researches indicated that intake of 500 g/d grape juice (~ 1066 mg polyphenol) [40] and GSE 0.15 g/d (~ 150 mg polyphenol) [41] or 0.6 g/d (~ 600 mg polyphenol) [42] significantly reduced the MDA levels. Although, intake of 90 g/d raisin (~ 178.75 mg polyphenol) had no significant effect on MDA levels [43]. SOD levels were significantly lower for subjects receiving 0.35 g/d whole grape extract (~ 350 mg polyphenol) compared to placebo group [1]. In the other study significant increasing and decreasing on SOD and TAC levels was observed respectively by intake of 0.2 g/d GSE (~ 200 mg polyphenol) [44]. Moreover, 0.1 g/d resveratrol supplementation (~ 100 mg polyphenol) could decrease on GPX level in healthy men [45]. Besides, intake of 0.6 g/d GSE (~ 600 mg polyphenol) [23] and 12 g/d grape powder (500 mg total polyphenol) [46] showed a significant increase on GSH.

Although several clinical trials investigated the effect of GPCP intake on oxidative stress markers, no conclusive result exists on this issue. Furthermore, no systematic review and meta-analysis has ever been conducted in this area. Therefore, this systematic review and meta-analysis aimed to investigate the effect of GPCP on oxidative stress.

## Materials and methods

### Search strategy

This systematic review and meta-analysis was conducted according to the PRISMA (Preferred Reporting Items for Systematic Reviews and Meta-Analyses Guidelines) [47].

The protocol of this study was also registered on PROSPERO, an International Prospective Register of Systematic Reviews (http://www.crd.york.ac.uk/PROSPERO) with the registration no of CRD42019116696.

We searched PubMed, Scopus, Web of Science, and Scholar up to August 20, 2020 using Medical Subject Heading terms (MeSH) and non-MeSH terms to evaluate the effect of GPCP supplementation on oxidative stress biomarkers. The following keywords were used in the search:

(grape* OR “grape polyphenol” OR “grape seed extract” OR “grape seed” OR “Grape Seed Proanthocyanidins” OR raisin* OR polyphenol* OR “V*itis vinifera*” OR raisin* OR “grape extract” OR wine* OR “grape polyphenols” OR “grape powder” OR “concord grape juice” OR “grape juice”) AND (“ oxidative stress “ OR “ Superoxide Dismutase” OR “ Superoxide Peroxidase “ OR “oxidative stress indices” OR “Glutathione Peroxidase” OR “ oxidative stress markers” OR “oxidative mediators” OR “oxidative biomarker” OR “F_2_-isoprostanes” OR “ isoprostanes “ OR malondialdehyde* OR MDA OR “Catalase” OR CAT OR “ total antioxidant capacity “ OR “ total antioxidant status” OR “total oxidant status “ OR TAS OR TOS OR Glutathione* OR TBARS OR “ Reduced Glutathione” OR “ Thiobarbituric Acid Reactive Substances “ OR ORAC OR “Oxygen Radical Absorbance Capacity “ OR TAC OR GPX OR SOD OR GSH) AND (trial* OR “randomized controlled trials” OR RCT OR “ Clinical Trials as Topic “ OR “clinical trials” OR “randomized controlled clinical trial” OR “randomized clinical trials” OR “controlled clinical trials” OR intervention OR Intervention OR randomized OR randomised OR random OR randomly OR placebo OR assignment OR cross-over OR parallel) NOT (animal* OR rat OR rats OR rabbit* OR rattus OR monkey* OR mice* OR mouse* OR hen* OR chicken* OR duck* OR pig* OR cow* OR “cell line” OR non-human OR “In-vitro” OR “In-vivo”). Furthermore, Reference lists of the related original and review articles were also carefully checked to obtain other eligible studies.

### Selection criteria

The inclusion criteria for the studies were: 1) having an RCT design; 2) evaluating the effect of GPCP on oxidative stress biomarkers versus placebo or other intervention, such as water or usual wine; 3) reporting the dose of GPCP; 4) having participants with 18 years of age or older, and 5) being in English.

### Study selection

**Fig. 1:**
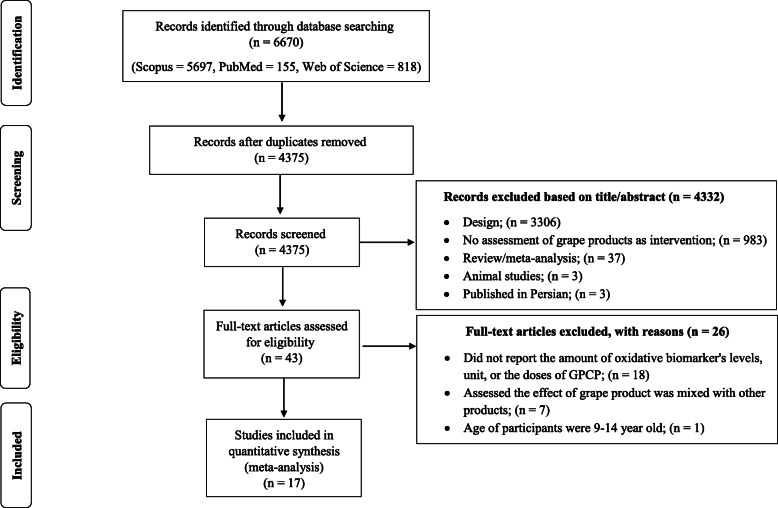
Study flow diagram depicting the review process following the Preferred Reporting Items for Systematic Reviews and Meta-Analyses

Two researchers separately performed the initial screening on the basis of the titles and abstracts of the articles. In the next step, the full texts of all related articles were investigated by reviewers to find studies over the effect of GPCP on oxidative stress. Eventually, any disagreements were discussed and resolved by consensus with third researcher (Fig. 1).

### Data extraction

Two independent researchers summarized the data on the studies’ author’s family name, publication year, sample size and rate of sample loss, dose and type of intervention, study duration, cross-over or parallel study design, gender, age and healthy status of participants, as well as mean and SD of oxidative stress biomarkers levels at the baseline and end of trial. The collected information was double-checked by a third researcher.

### Quality assessment

At this stage, two reviewers independently evaluated the methodological quality of the eligible studies through Cochrane Collaboration’s tool including six domains of: 1) random sequence generation (selection bias); 2) allocation concealment (selection bias); 3) blinding of participants and personnel (performance bias); 4) blinding of outcome assessment (detection bias); 5) incomplete outcome data (attrition bias); and 6) selective reporting (reporting bias). Each domain was classified to three categories: low risk of bias, high risk of bias and unclear risk of bias. According to the mentioned domains, the overall quality of each study was considered as good (low risk for more than 2 item), fair (low risk for 2 item), or weak (low risk for less than 2 item) [48].

### Data synthesis and analysis

Statistical analyses were conducted using STATA software, version 11.2 (STATA Corp, College Station, TX). The random effects model which takes the between-study heterogeneity into account was used to calculate the weighted mean difference (WMD) and its 95% confidence intervals (CIs) [49]. To incorporate between-study variation, a random effects model was also applied to combine effect sizes. This model takes between-study heterogeneity into account. To evaluate heterogeneity among studies, I^2^ and Q statistic were used. If I^2^ > 50% and *p*-value of Q statistic < 0.05, statistically significant heterogeneity was recognized [50]. Subgroup analysis was conducted to explore the possible source of heterogeneity among the studies for all of oxidative biomarkers were addressed in our meta-analysis. Subgroup analysis included the following: duration, grape polyphenol doses, study design (parallel and crossover), study quality (weak, fair, good), and health status of study population (healthy individuals: people with no clinical condition versus unhealthy individuals: overweight; obese; chronic obstructive pulmonary disease; chronic kidney disease; coronary artery disease or ≥ 1 cardiac risk factor; type 2 diabetes).

Publication bias was assessed by examination of the funnel plot and formal testing for “funnel plot” asymmetry using Begg’s test and Egger’s test [51]. Sensitivity analysis was performed to identify whether a specific study or a particular group of studies affected the outcomes [51]. *P* values of less than 0.05 were considered significant.

### Meta-regression

Meta-regression was conducted to evaluate the association of estimated effect size with dose and duration of the GPCP intake.

## Results

### Literature search

Our search in the databases of Google Scholar, PubMed, Web of Science, and Scopus resulted in 6670 articles. After removing the duplicate studies 4375 papers remained. Later after screening the included articles’ tittles and abstracts, 4332 other studies were excluded since they hadn’t RCT design (*n* = 3306), didn’t evaluate the effect of grape products as interventions (*n* = 983) and they were animal studies (n = 3), in Persian (n = 3). Review/meta-analysis studies (*n* = 37) with topics that appeared not to be related to our research question for example they addressed effects of dietary polyphenols [52–59], polyphenol-rich interventions [54, 60], antioxidant supplements [61], flavonoids [62, 63], resveratrol [64–67], alcoholic beverage [68–77], grape products [78–87], or fruit and vegetable juices consumption [88] which did not address our objects linking to oxidative stress biomarkers. However, reference lists of the related review articles were also carefully checked to obtain eligible studies. Later, full texts of the selected studies were reviewed and 26 papers were excluded since: they did not report the amount of oxidative biomarker’s levels [89–104], unit [105], or the dose of GPCP [106], assessed the effect of grape product along with other fruits [107–112] or drug [113], the age of participants were 9–14 year old [41]. Finally, 17 studies were included in our systematic review and meta-analysis **(**Fig. 1**).**

### Study characteristics

Characteristics of all studies that entered our systematic review and meta-analysis are indicated in Table 1. All studies were published from 2006 to 2018. The total number of included participants was 633 (intervention group: *N* = 376, control group: *N* = 355). The articles were conducted in Europe [43, 114–118], America [1, 37–39, 45, 119], and Asia [32, 40, 42, 44]. All studies had a randomized controlled trial with parallel [1, 32, 37, 40, 42–45] or cross over design [36, 38, 39, 114–118]. The trials lasted from 2 to 16 weeks and the dose of GPCP ranged from 0.1 g to 500 g. GPCPs were also administered in different forms such as [42, 44, 119], grape extract [1, 116], grape powder [38], juice [40], raisin [39, 43], resveratrol [36, 45], red wine [114, 115, 117, 118]. Considering the Cochrane risk of bias, 8, 2, and 7 articles had good, poor and fair quality, respectively (Table 2).

**Table 1:** Study design and participant characteristics of trials included in meta-analysis^a^

Study.Year		(Lu et al., 2018) [32]	(Kanellos et al., 2017) [43]	(Taghizadeh et al., 2016) [42]	(Saldanha et al., 2016) [36]	(Torres et al., 2015) [118]	(Macedo et al., 2015) [45]	(Zunino et al., 2014) [38]	(Evans et al., 2014) [1]	(Amoutzopoulos et al., 2013) [40]	(Noguer et al., 2012) [117]	(Pourghassem-Gargari et al., 2011) [44]	(Mellen et al., 2010) [119]	(Estruch et al., 2011) [115]	(Lafay et al., 2009) [116]	(Hollis et al., 2009) [37]	(Rankin et al., 2008) [39]	(Avellone et al., 2006) [114]
Product of Intervention group		OPCs	Raisin	GSE	Resveratrol	Red wine	Resveratrol	Grape Powder	WGE	grape-based beverage (hardaliye)	LPD + dealcoholized wine	GSE	GSE	Red wine	Grape extract	Concord grape juice	Raisin	Red wine
Country		Taiwan	Greece	Iran	Brazil	Spain	Brazil	USA	Canada	Turkey	Spain	Iran	USA	Spain	France	USA	USA	Italy
Study design		Parallel	Parallel	Parallel	Cross-over	Cross-over	Parallel	Cross-over	Cross-over	Parallel	Cross-over	Parallel	Cross-over	Cross-over	Cross-over	Parallel	Cross-over	Cross-over
Sample Size (I/C)		13/14	22/14	20/20	9/11	16/16	30/30	24/24	13/12	39/17	8/8	26/22	50/50	20/20	20/20	25/26	17/17	24/24
Health status of participants		COPD patient	Healthy smokers	Healthy subjects	CKD patient	Healthy subjects	Healthy subjects	Healthy obese	prehypertensive, overweight	Healthy subjects	Healthy subjects	T2D	CAD or ≥ 1 cardiac risk factor	Healthy subjects	Healthy subjects	Healthy subjects	Over weight	Healthy subjects
Age of participants^b^, y (I/C)		71 ± 7.2 / 71 ± 7.4	30.8 ± 7.5/29.8 ± 5.23	19.8 ± 5 /21.6 ± 7	63.0 ± 7.5/62.0 ± 8.4	Total: 25.5	21.46 ± 9.68 /22.3 ± 9.73	Men: 37.1 ± 10.5 Women: 34.7 ± 13.9	46.1 ± 11.1 /38.0 ± 12.3	37.44 ± 8.33 /34.18 ± 5.96	Total: 28 ± 5.3	Total: 47.5	Total: 52.1 ± 8.1	Total: 37.6 ± 7.4	Total: 21.6 ± 8.9	22.0 ± 4.0/26.0 ± 9.0	Total: 26.5 ± 7.6	Total: 43 ± 10.6
BMI of participants^b^, Kg/m^2^ (I/C)		NR	24.4 ± 2.81/24.4 ± 2.99	21.6 ± 3.6/21.2 ± 1.5	26.8 ± 5.6/28.6 ± 4.4	NR	NR	Men: 36.6 ± 4.4 Women: 36.9 ± 5.3	Total:29.95	24.81 ± 3.29/24.97 ± 3.04	NR	31.0 ± 6.0/30.0 ± 4.0	Total: 29.8 ± 6.0	NR	Total: 23.9 ± 2.4	27.0 ± 1.6 /27.0 ± 1.5	Total: 33.5 ± 6.7	Total: 23 ± 2.5
Sex of participants	**M**	NR	27	0	9	8	60	8	12	34	NR	NR	25	40	20	NR	8	28
	**F**	NR	9	40	11	8	0	16	14	22	NR	NR	25	0	0	NR	9	20
Product of Control group		Placebo	No raisin	Placebo	Placebo	Water with sugar	Placebo	Placeb Powder	Placebo	No hardaliye	LPD	Placebo	Placebo	Gin	Placebo	Polyphenol free grape flavored drink	Placebo	Usual wine
Dose of grape product, g/d		0.15	90	0.6	0.5	160	0.1	92	0.35	500	300	0.2	1.3	320	0.4	480	90	250
Dose of GPP, mg/d		~ 150	178.75	~ 600	~ 500	361.6	~ 100	62.24	~ 350	1066	~ 780	~ 200	~ 1300	~ 832	~ 400	~ 945.5	~ 178.75	~ 650
Period, wk		8	4	8	16	8	12	9	6	6	2	8	14	12	10	12	8	12
SOD^b^, U/mgHb	I	0.0061 ± 0.003	NR	NR	0.0037 ± 0.002	NR	0.005 ± 0.002	NR	0.004 ± 0.0007	NR	0.04 ± 0.01	0.001 ± 0.0002	NR	0.001 ± 0.0002	0.0025 ± 0.0004	NR	NR	NR
	C	0.0068 ± 0.003	NR	NR	0.0039 ± 0.002	NR	0.006 ± 0.003	NR	0.005 ± 0.002	NR	0.05 ± 0.01	0.001 ± 0.0001	NR	0.001 ± 0.0001	0.0023 ± 0.0003	NR	NR	NR
TAC^b^, mmol/L	I	NR	NR	811.4 ± 122.9	NR	19.3 ± 2.4	NR	NR	1.7 ± 1.14	0.23 ± 0.07	NR	NR	0.6 ± 0.07	NR	NR	NR	NR	0.9 ± 0.02
	C	NR	NR	826.5 ± 128.9	NR	17.7 ± 1.5	NR	NR	1.2 ± 0.8	0.25 ± 0.1	NR	NR	0.6 ± 0.07	NR	NR	NR	NR	1 ± 0.02
ORAC^b^, μmol/L	I	NR	NR	NR	NR	NR	NR	1536.14 ± 164.44	NR	NR	2586 ± 473	NR	NR	NR	13,885 ± 1609.2	2093 ± 827	7163 ± 1513	NR
	C	NR	NR	NR	NR	NR	NR	1536.14 ± 164.44	NR	NR	2657 ± 406	NR	NR	NR	14,488 ± 1698.6	2555 ± 936	8335.7 ± 1760.6	NR
MDA^b^, μmol/L	I	7.1 ± 1.3	4.3 ± 1.4	4.7 ± 2.1	NR	NR	NR	NR	NR	1.5 ± 0.3	NR	3.5 ± 0.6	0.5 ± 0.2	0.041 ± 0.012	NR	NR	NR	NR
	C	7.9 ± 1.5	5.1 ± 2.2	3.9 ± 1.1	NR	NR	NR	NR	NR	1.6 ± 0.4	NR	3.5 ± 0.7	0.5 ± 0.2	0.049 ± 0.017	NR	NR	NR	NR
GPX^b^, U/mgHb	I	NR	NR	NR	0.0008 ± 0.0003	NR	0.097 ± 0.037	NR	NR	NR	NR	0.029 ± 0.003	NR	0.0023 ± 0.0005	0.018 ± 0.004	NR	NR	NR
	C	NR	NR	NR	0.0007 ± 0.0003	NR	0.123 ± 0.036	NR	NR	NR	NR	0.022 ± 0.002	NR	0.0022 ± 0.0006	0.017 ± 0.004	NR	NR	NR

^a^ Meta-analyses were conducted with the use of the random-effects model. Main analysis: all studies from Table 1, includes both no-grape polyphenols controls and grape product contain polyphenols intake. *BMI* Body Mass Index, *C* Control group, *CAD* Coronary artery disease, *CKD* Chronic Kidney Disease, *COPD* Chronic Obstructive Pulmonary Disease, *F* Female, *GPP* Grape polyphenols, *GPX* Glutathione Peroxidase, *GSE* Grape Seed Extract, *I* Intervention group, *LPD* Low phenolic diet, *M* Male, *MDA* malondialdehyde, *NR* Not Reported, *OPCs* Oligomeric proanthocyanidins extracted from grape seeds, *ORAC* Oxygen Radical Absorbance Capacity, *SOD* Superoxide Dismutase, *TAC* Total Antioxidant Capacity, *T2D* Type 2 diabetes, *USA* United States of America, *WGP* Whole grape extract

^b^ Values are mean ± SDs

**Table 2:** Risk of bias assessment for included randomized controlled clinical trials

Study	Random sequence generatin (Selection bias)	Allocation concealment (Selection bias)	Blinding of participants and personnel (Performance bias)	Blinding of outcome assessment (Detection bias)	Incomplete outcome data (Attrition bias)	Selective reporting (Reporting bias)	Overall quality
**Lu et al.** **[**32**]**	Unclear	Unclear	Low	Unclear	Unclear	Low	Fair
**Kanellos et al.** **[**43**]**	Low	Low	Low	Low	Unclear	Low	Good
**Saldanha et al.** **[**36**]**	Low	Low	Low	Low	Low	Low	Good
**Taghizadeh et al.** **[**42**]**	Low	Low	Low	Unclear	Low	Low	Good
**Torres et al.** **[**118**]**	Unclear	Unclear	Unclear	Unclear	low	Low	Fair
**Macedo et al.** **[**45**]**	Unclear	Unclear	Low	Unclear	Unclear	Low	Fair
**Zunino et al.** **[**38**]**	Unclear	Unclear	Low	Unclear	Low	Low	Good
**Evans et al.**	Low	Unclear	Low	Unclear	Low	Low	Good
**Amoutzopoulos et al.**	Unclear	Unclear	Unclear	Unclear	Low	Low	Fair
**Noguer et al.** **[**117**]**	Unclear	Unclear	Unclear	Unclear	Unclear	Low	Weak
**Pourghassem et al.** [44]	Unclear	Unclear	Unclear	Unclear	Low	Low	Fair
**Mellen et al.** **[**119**]**	Unclear	Unclear	Low	Unclear	Low	Low	Good
**Estruch et al .[**115**]**	Low	Unclear	Unclear	Unclear	Low	Low	Good
**Lafay et al.** **[**116**]**	Unclear	Unclear	Low	Unclear	Unclear	Low	Good
**Hollis et al.** **[**37**]**	Unclear	Unclear	Unclear	Unclear	Low	Low	Fair
**Rankin et al.** **[**39**]**	Unclear	Unclear	Unclear	Unclear	Low	Low	Fair
**Avellone et al.** **[**114**]**	Unclear	Unclear	Unclear	Unclear	Unclear	Low	Weak

### Effect of GPCP on SOD levels

**Fig. 2:**
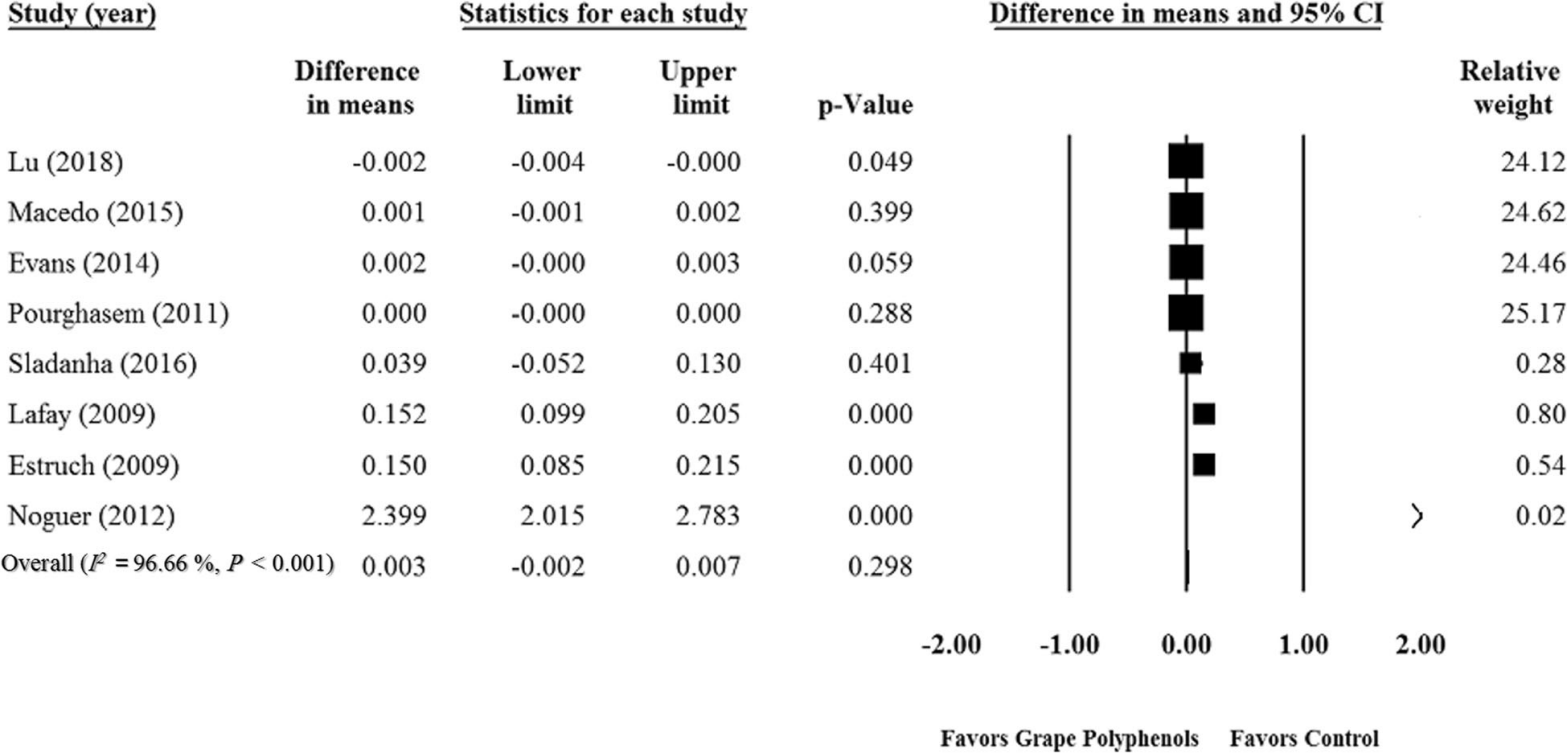
Forest plot of the effect of grape products containing polyphenols (GPCP) on superoxide dismutase levels

According to the data meta-analysis, some studies [1, 32, 36, 44, 45, 115–117] showed that GPCP had no significant effect on SOD levels (WMD = 0.003 U/mgHb; 95% CI: − 0.002, 0.007; *P* = 0.29) (Fig. 2). This finding did not change after sensitivity analysis Supplementary Fig. 1), but a significant heterogeneity was observed among the studies (*P* <  0.001, *I*^*2*^ = 96.66). Although funnel plots showed publication bias for the related studies (Supplementary Fig. 2), asymmetry tests showed no publication bias (Begg’s test, *P* = 0.10 and Egger‘s test, *P* = 0.09). According to the subgroup analysis, intake of ≥400 mg/d grape polyphenol doses had a significant effect on SOD level (Dose < 400 mg/d: WMD < 0.001 U/mgHb; 95% CI: − 0.001, 0.001; *P* = 0.75; ≥ 400 mg/d: WMD = 0.539 U/mgHb; 95% CI: 0.24, 0.82; P < 0.001) (Table 3). The impact of supplementation with GPCP was significant only among the healthy participants (Healthy individuals: WMD = 0.450 U/mgHb; 95% CI: 0.23, 0.66; P < 0.001; Unhealthy participants: WMD < 0.001 U/mgHb; 95% CI: − 0.001, 0.001; *P* = 0.99) (Table 3). Furthermore, GPCP had no significant effect on SOD in supplementation durations of ≥10 weeks versus < 10 weeks (Duration < 10 wk.: WMD = 0.001 U/mgHb; 95% CI: − 0.006, 0.007; *P* = 0.86; Duration ≥10 wk.: WMD = 0.085 U/mgHb; 95% CI: − 0.01, 0.18; *P* = 0.09) (Table3). Cross-over studies (Cross-over: WMD = 0.539 U/mgHb; 95% CI: 0.24, 0.82; P < 0.001; Parallel: WMD < 0.001 U/mgHb; 95% CI: − 0.001, 0.001; *P* = 0.75) (Table 3) and poor quality studies indicated a significant effect on SOD (Good quality: *n* = 4, WMD = 0.085 U/mgHb; 95% CI: − 0.01, 0.18; *P* = 0.08; Fair quality: *n* = 3, WMD < -0.001 U/mgHb; 95% CI: − 0.001, 0.001; P = 0.75; Weak quality: *n* = 1, WMD = 2.399 U/mgHb; 95% CI: 2.01, 2.78; P < 0.001) (Table 3). Meta-regression analysis also showed a significant association between the administered dose of GPCP and SOD concentrations (slope = 0.00001; 95% CI: < 0.00001, 0.00002; *P* = 0.034), while the GPCP dosage had no significant relationship with the supplementation duration (slope = 0.00002; 95% CI: − 0.00031, 0.00035; *P* = 0.924) (Supplementary Figs. 3 A, B).

**Table 3:** Subgroup analysis to assess the effect of grape polyphenols supplementation on different biomarkers of oxidative stress levels^a^

Serum enzyme	Number of trials	WMD	95% CI (upper limit, lower limit)	*P* value
SOD				
Health status				
Unhealthy	4	< 0.001	(−0.001, 0.001)	0.99
Healthy	4	0.450	(0.23, 0.66)^b^	< 0.001
Duration of study				
10 wk. or more	4	0.085	(−0.01, 0.18)^b^	0.09
lower than 10 wk	4	0.001	(−0.006, 0.007)	0.86
Dose of grape polyphenols				
400 mg/d or more	4	0.539	(0.24, 0.82)^b^	< 0.001
Lower than 400 mg/d	4	< 0.001	(−0.001, 0.001)	0.75
Type of study				
Cross-over	4	0.539	(0.24, 0.82)^b^	< 0.001
Parallel	4	< 0.001	(−0.001, 0.001)	0.75
**Study quality**				
Good	4	0.085	(−0.01, 0.18)	0.08
Fair	3	< -0.001	(−0.001, 0.001)	0.75
Weak	1	2.399	(2.01, 2.78)^b^	< 0.001
TAC				
Health status				
Unhealthy	2	−0.254	(−0.65, 0.14)	0.21
Healthy	4	2.829	(0.13, 5.52)^b^	0.04
Duration of study				
More than 6 wk	3	3.814	(−4.14, 11.77)	0.34
6 wk. or lower	3	0.084	(−0.36, 0.52)	0.71
Dose of grape polyphenols				
More than 650 mg/d	2	−0.058	(−0.22, 0.11)	0.49
650 mg/d or lower	4	2.595	(−2.53, 7.72)	0.32
Type of study				
Cross-over	3	2.785	(0.01, 5.55)^b^	0.04
Parallel	3	−0.164	(−0.65, 0.33)	0.51
**Study quality**				
Good	3	−0.145	(−1.18, −0.10)^c^	< 0.001
Fair	2	0.266	(−0.22, 0.75)	0.28
Weak	1	8.01	(7.54, 8.49)^b^	< 0.001
ORAC				
Health status				
Abnormal wt.^d^	2	−0.030	(−0.46, 0.40)	0.89
Healthy	3	0.524	(0.42, 0.62)^b^	< 0.001
Duration of study				
10 wk. or more	2	0.543	(0.43, 0.64)^b^	< 0.001
Lower than 10 wk	3	0.089	(−0.26, 0.44)	0.62
Dose of grape polyphenols				
More than 400 mg/d	2	0.377	(0.08, 0.67)^b^	0.01
400 mg/d or lower	3	0.161	(−0.26, 0.58)	0.46
Type of study				
Cross-over	4	0.210	(−0.15, 0.57)	0.25
Parallel	1	−140.0	(−651.9, 371.9)	0.59
**Study quality**				
Good	2	0.365	(0.01, 0.71)^b^	0.03
Fair	2	− 0.251	(−0.36, −0.13)^c^	< 0.001
Weak	1	0.377	(0.08, 0.67)^b^	0.01
MDA				
Health status				
Unhealthy	3	− 0.092	(−0.50, 0.32)	0.66
Healthy	4	−0.214	(−0.62, 0.19)	0.30
Duration of study				
8 wk. or more	5	−0.149	(−0.47, 0.17)	0.36
Lower than 8 wk.	2	0.096	(−1.14, 1.33)	0.88
Dose of grape polyphenols				
600 mg/d or more	4	−0.237	(−0.58, 0.11)	0.18
Lower than 600 mg/d	3	−0.034	(−0.88, 0.81)	0.93
Type of study				
Cross-over	2	0.003	(−0.05, 0.05)	0.90
Parallel	5	−0.328	(−0.79, 0.14)	0.17
**Study quality**				
Good	4	−0.072	(−0.55, 0.40)	0.77
Fair	3	−0.313	(−0.67, 0.05)	0.09
GPX				
Health status				
Unhealthy	2	0.009	(−0.02, 0.04)	0.60
Healthy	3	0.044	(−0.03, 0.12)	0.30
Duration of study				
More than 10 wk	3	−0.002	(−0.01, 0.01)	0.83
10 wk. or lower	2	0.068	(−0.07, 0.20)	0.33
Dose of grape polyphenols				
400 mg/d or more	3	0.063	(−0.02, 0.15)	0.16
Lower than 400 mg/d	2	< 0.001	(−0.001, 0.001)	0.80
Type of study				
Cross-over	3	0.063	(−0.02, 0.15)	0.16
Parallel	2	< 0.001	(−0.001, 0.001)	0.80
**Study quality**				
Good	3	0.063	(−0.02, 0.15)	0.16
Fair	2	< 0.001	(−0.001, 0.001)	0.80

^a^ *CI* confidence interval, *GPX* Glutathione Peroxidase, *MDA* malondialdehyde, *ORAC* Oxygen Radical Absorbance Capacity, *SE* standard error, *SOD* Superoxide Dismutase, *TAC* Total Antioxidant Capacity, *WMD* weighted mean difference, *wt* weight

^b^ Significant increase in the outcome was observed

^c^ Significant decrease in the outcome was observed

^d^ Abnormal weight that is, participants were obese or overweight

### Effect of GPCP on TAC levels

**Fig. 3:**
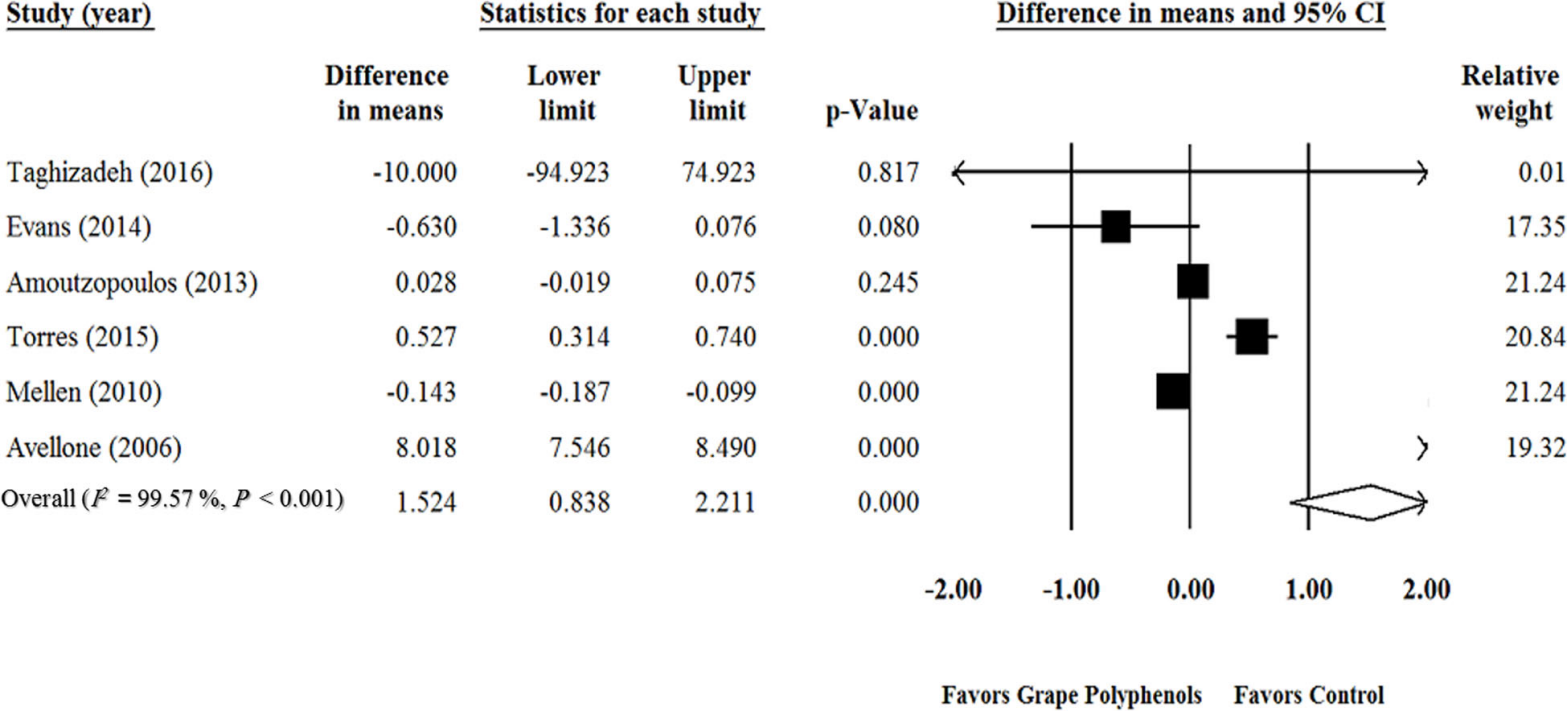
Forest plot of the effect of grape products containing polyphenols (GPCP) on total antioxidant capacity (TAC) levels

Meta-analysis of six RCTs [1, 40, 42, 114, 118, 119] showed that GPCP supplementation increased the TAC levels significantly (WMD = 1.524 mmol/L; 95% CI: 0.83, 2.21; *P* < 0.001) (Fig. 3). This result remained significant in sensitivity analysis) supplementary Fig. 4). A significant heterogeneity was observed among the studies (*P* <  0.001, *I*^*2*^ = 99.57). No publication bias was determined based on the Funnel plot and symmetry tests (Begg’s test, *p* = 0.70; Egger‘s test, *P* = 0.28) among the related studies (Supplementary Fig. 5). According to the subgroup analysis, duration of study (Duration ≤6 wk.: WMD = 0.084 mmol/L; 95% CI: − 0.36, 0.52; *P* = 0.71; Duration > 6 wk.: WMD = 3.814 mmol/L; 95% CI: − 4.14, 11.77; *P* = 0.34) (Table 3) and grape polyphenol dose (Dose ≤650 mg/d: WMD = 2.595 mmol/L; 95% CI: − 2.53, 7.72; *P* = 0.32; Dose > 650 mg/d: WMD = − 0.058 mmol/L; 95% CI: − 0.22, 0.11; *P* = 0.49) had no significant impact on TAC (Table 3). The GPCP supplementation significantly increased TAC only among the healthy participants (healthy subjects: WMD = 2.829 mmol/L; 95% CI: 0.13, 5.52; *P* = 0.04; unhealthy subjects: WMD = − 0.254 mmol/L; 95% CI: − 0.65, 0.14; *P* = 0.21) (Table 3). The cross-over type of study had a significant effect on TAC (Cross-over: WMD = 2.785 mmol/L; 95% CI: 0.01, 5.55; P = 0.04; Parallel: WMD = − 0.164 mmol/L; 95% CI: − 0.65, 0.33; *P* = 0.51) (Table 3). Moreover, poor and good quality studies had significant effects on TAC (Good quality: *n* = 3, WMD = − 0.145 mmol/L; 95% CI: − 1.18, − 0.10; P < 0.001; Fair quality: *n* = 2, WMD = 0.266 mmol/L; 95% CI: − 0.22, 0.75; *P* = 0.28; Weak quality: *n* = 1, WMD = 8.018 mmol/L; 95% CI: 7.54, 8.49; P < 0.001) (Table 3). The duration (slope = − 0.02; 95% CI: − 0.02, − 0.01; P < 0.00001) and dose of GPCP supplementation (slope = − 0.001; 95% CI: − 0.0014, − 0.0010; P < 0.00001) had a significant association with TAC in meta-regression analysis (Supplementary Figs. 6 A, B).

### Effect of GPCP on ORAC levels

**Fig. 4:**
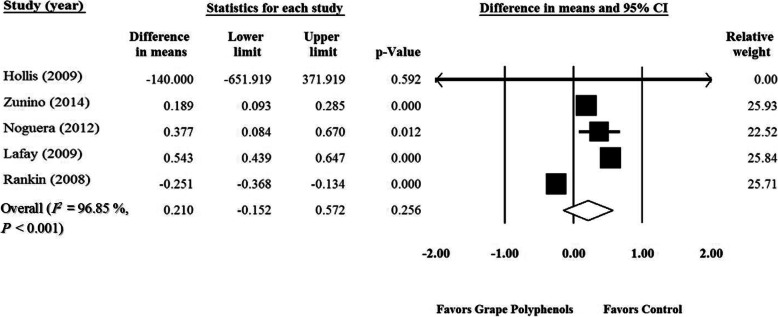
Forest plot of the effect of grape products containing polyphenols (GPCP) on oxygen radical absorbance capacity levels

Meta-analysis of five RCTs [37–39, 116, 117] demonstrated no significant effect of GPCP on ORAC levels (WMD = 0.210 μmol/L; 95% CI: − 0.15, 0.57; *P* = 0.25) (Fig. 4). This result did not change after the sensitivity analysis) Supplementary Fig. 7). A significant heterogeneity was seen among the studies (P < 0.001, *I*^*2*^ = 96.02). No significant publication bias was found with regard to the related studies based on the funnel plots (Supplementary Fig. 8) and asymmetry tests (Begg’s test, *P* = 0.80 and Egger‘s test, *P* = 0.94). According to the subgroup analysis, intake of GPCP had a significant effect on healthy individuals (Healthy participants: WMD = 0.524 μmol/L; 95% CI: 0.42, 0.62; P < 0.001; Abnormal wt. subjects: WMD = − 0.03 μmol/L; 95% CI: − 0.46, 0.40; *P* = 0.89) (Table 3). Higher durations and grape polyphenol doses of GPCP supplementation had a significant impact on ORAC (Duration < 10 wk.: WMD = 0.089 μmol/L; 95% CI: − 0.26, 0.44; *P* = 0.62; Duration ≥10 wk.: WMD = 0.543 μmol/L; 95% CI: 0.43, 0.64; P < 0.001) (Dose ≤400 mg/d: WMD = 0.161 μmol/L; 95% CI: − 0.26, 0.58; *P* = 0.46; Dose > 400 mg/d: WMD = 0.377 μmol/L; 95% CI: 0.08, 0.67; *P* = 0.01) (Table 3). Supplementation with GPCP had no significant effect on ORAC in parallel and cross-over studies (Cross-over: WMD = 0.210 μmol/L; 95% CI: − 0.15, 0.57; P = 0.25; Parallel: WMD = − 140.0 μmol/L; 95% CI: − 651.9, 371.9; *P* = 0.59) (Table 3). Moreover, poor, fair, and good quality studies had a significant effect on ORAC (Good quality: *n* = 2, WMD = 0.365 μmol/L; 95% CI: 0.01, 0.71; *P* = 0.03; Fair quality: n = 2, WMD = − 0.251 μmol/L; 95% CI: − 0.36, − 0.13; P < 0.001; Weak quality: *n* = 1, WMD = 0.377 μmol/L; 95% CI: 0.08, 0.67; P = 0.01) (Table 3). The dose (slope = 0.0008; 95% CI: 0.0005, 0.0012; P < 0.0001), and duration of GPCP supplementation (slope = 0.069; 95% CI: 0.03, 0.10; *P* = 0.0002) (Supplementary Figs. 9 A, B) indicated a significant association with ORAC in meta-regression analysis.

### Effect of GPCP on MDA levels

**Fig. 5:**
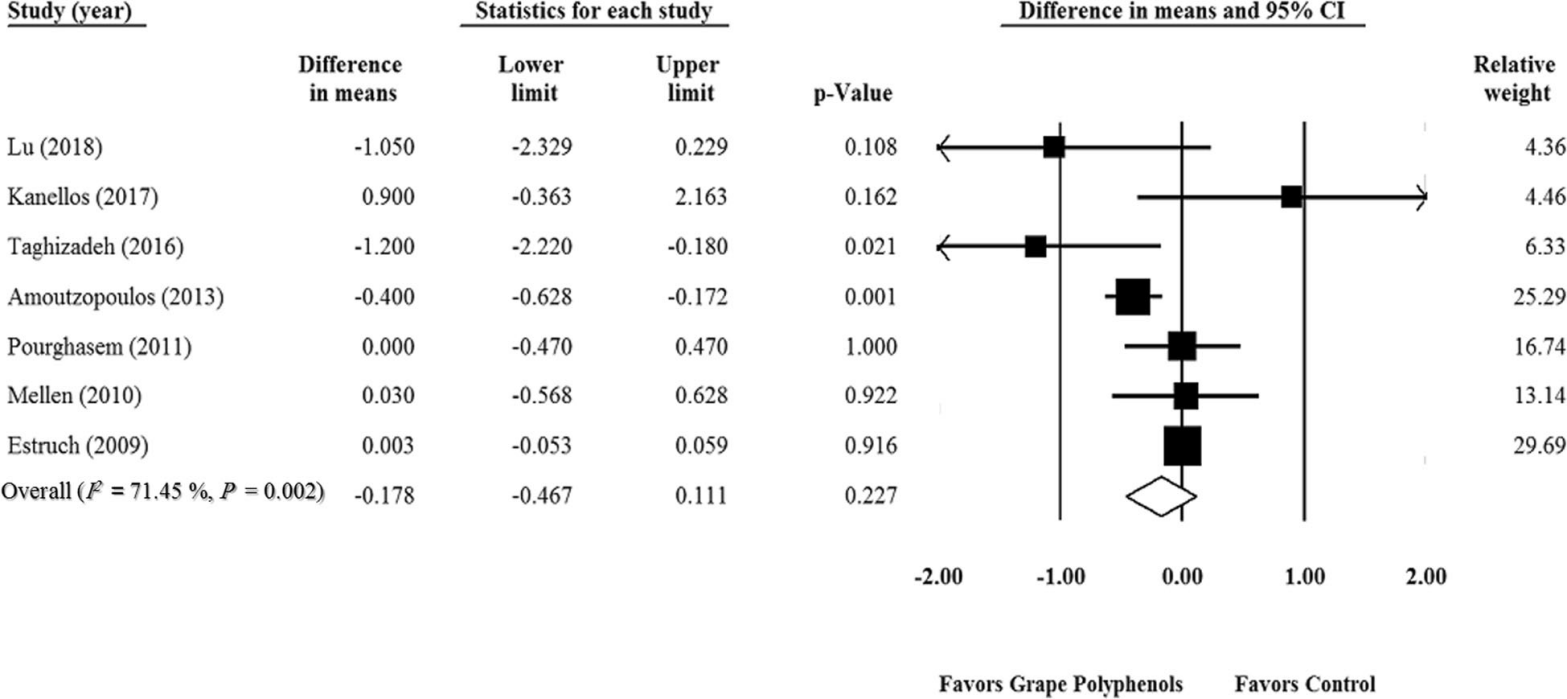
Forest plot of the effect of grape products containing polyphenols (GPCP) on Malondialdehyde levels

According to the meta-analysis of seven RCTs [32, 40, 42–44, 115, 119], GPCP had no significant impact on MDA levels (WMD = − 0.178 μmol/L; 95% CI: − 0.46, 0.11; *P* = 0.22) (Fig. 5). This finding did not change after the sensitivity analysis) Supplementary Fig. 10). A significant heterogeneity was observed among the studies (*P* = 0.002, *I*^*2*^ = 71.454). Funnel plots and asymmetry tests indicated no publication bias in the related studies (Begg’s test, *P* = 1.00 and Egger‘s test, *P* = 0.30) (Supplementary Fig. 11). The findings of subgroup analysis demonstrated no significant effect of GPCP on MDA in healthy and unhealthy participants (Healthy subjects: WMD = − 0.214 μmol/L; 95% CI: − 0.62, 0.19; P = 0.30; Unhealthy participants: WMD = − 0.092 μmol/L; 95% CI: − 0.50, 0.32; *P* = 0.66) (Table 3). Moreover, GPCP impact was not significant with regard to different supplementation durations (Duration < 8 wk.: WMD = 0.096 μmol/L; 95% CI: − 1.14, 1.33; *P* = 0.88; Duration ≥8 wk.: WMD = − 0.149 μmol/L; 95% CI: − 0.47, 0.17; *P* = 0.36) and grape polyphenol doses (Dose < 600 mg/d: WMD = − 0.034 μmol/L; 95% CI: − 0.88, 0.81; *P* = 0.93; Dose ≥600 mg/d: WMD = − 0.237 μmol/L; 95% CI: − 0.58, 0.11; *P* = 0.18) (Table 3). Parallel and cross-over types of study (Cross-over: WMD = 0.003 μmol/L; 95% CI: − 0.05, 0.05; *P* = 0.90; Parallel: WMD = − 0.328 μmol/L; 95% CI: − 0.79, 0.14; *P* = 0.17) (Table 3) as well as Quality of studies (Good quality: *n* = 4, WMD = − 0.072 μmol/L; 95% CI: − 0.55, 0.40; *P* = 0.77; Fair quality: *n* = 3, WMD = − 0.313 μmol/L; 95% CI: − 0.67, 0.05; *P* = 0.09) (Table 3) had no significant effect on MDA. Meta-regression analysis showed that MDA levels had a significant association with GPCP supplementation duration (slope = 0.05; 95% CI: 0.022, 0.094; *P* = 0.001), but this relationship was not significant with the GPCP supplementation dose (slope = − 0.0003; 95% CI: − 0.0008, 0.00001; P = 0.17) (Supplementary Figs. 12 A, B).

### Effect of GPCP on GPX levels

**Fig. 6:**
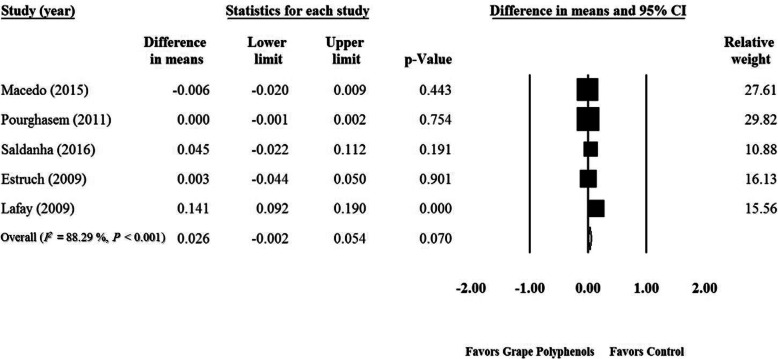
Forest plot of the effect of grape products containing polyphenols (GPCP) on *glutathione peroxidase* levels

Meta-analysis of five RCTs [36, 44, 45, 115, 116] showed no significant effect of GPCP on GPX levels (WMD = 0.026 U/mgHb; 95% CI: − 0.002, 0.05; *P* = 0.07) (Fig. 6). This finding remained unchanged after the sensitivity analysis (Supplementary Fig. 13). A significant heterogeneity was observed among the studies (P < 0.0001, *I*^*2*^ = 88.29). Although the funnel plots indicated a publication bias in the studied surveys (Supplementary Fig. 14), asymmetry tests did not confirm this result (Begg’s test, *P* = 0.08 and Egger‘s test, *P* = 0.33). According to the subgroup analysis, GPCP had no significant effect among healthy and unhealthy participants (Healthy participants: WMD = 0.044 U/mgHb; 95% CI: − 0.03, 0.12; *P* = 0.30; Unhealthy participants: WMD = 0.009 U/mgHb; 95% CI: − 0.02, 0.04; *P* = 0.60) (Table 3). Similarly, GPCP had no significant impact on GPX in various supplementation durations (Duration ≤10 wk.: WMD = 0.068 U/mgHb; 95% CI: − 0.07, 0.20; P = 0.33; Duration > 10 wk.: WMD = − 0.002 U/mgHb; 95% CI: − 0.01, 0.01; *P* = 0.83) and grape polyphenol doses (Dose < 400 mg/d: WMD < 0.001 U/mgHb; 95% CI: − 0.001, 0.001; *P* = 0.80; Dose ≥400 mg/d: WMD = 0.063 U/mgHb; 95% CI: − 0.02, 0.15; *P* = 0.16) (Table 3). Parallel and cross-over types of study (Cross-over: WMD = 0.063 U/mgHb; 95% CI: − 0.02, 0.15; P = 0.16; Parallel: WMD < 0.001 U/mgHb; 95% CI: − 0.001, 0.001; P = 0.80) (Table 3) as well as quality of studies (Good quality: *n* = 3, WMD = 0.063 U/mgHb; 95% CI: − 0.02, 0.15; P = 0.16; Fair quality: *n* = 2, WMD < 0.001 U/mgHb; 95% CI: − 0.001, 0.001; P = 0.80) had no significant effect on the GPX level (Table 3). The trial duration (slope = 0.0009; 95% CI: − 0.002, 0.004; *P* = 0.58) and GPCP dose (slope = 0.00007; 95% CI: 0.00001, 0.00013; *P* = 0.027) (Supplementary Figs. 15 A, B) showed no significant relationship with the effect of GPCP supplementation on GPX in meta-regression analysis.

## Discussion

Findings showed that GPCP intake had a significant effect on increasing the TAC levels; however, it had no significant impact on other oxidative stress biomarkers. In the sub-group analysis, GPCP significantly increased SOD, TAC, and ORAC levels in healthy participants. Furthermore, higher GPCP doses increased ORAC and SOD levels significantly. Longer intervention periods also increased the ORAC levels. Finally, cross-over study design enhanced the TAC and SOD levels.

To the best of our knowledge, this systematic review and meta-analysis is the first study over the effect of supplementation with GPCP on oxidative stress. In the same line with our findings, other systematic reviews and meta-analyses confirmed the beneficial effects of GPCP on the obesity-induced chronic inflammation [120], lipid profile [121], blood glucose [87], systolic blood pressure, heart rate [57, 122], endothelial function [56], liver and heart functions [87], metabolic syndrome, and type 2 diabetes [120]. In addition, the significant impact of GPCP was reported on oxidative stress in patients with Type 2 diabetes [23, 44], pre hypertension [1, 123], overweight [39], acute lymphoblastic leukemia [41], CVD [100, 124], systemic sclerosis [96], Chronic Obstructive Pulmonary Disease [32], hemodialysis patient [46, 125, 126], hyperlipidemia [127], smoker [128], and healthy subjects [21, 26, 40, 42, 90, 92, 94, 101, 102, 105, 114, 115, 118]. In contrast with our results, some RCTs [36, 38, 43, 95, 104, 106, 116, 119, 129] reported no significant effect of GPCP on oxidative stress. The doses and duration of supplementation with GPCP [36], co-administration of minerals and vitamins [36], polyphenols consumption from foods [117], physical activity [130, 131], and age of participants [132] adipose tissue [91], baseline oxidative stress levels [36], and other individual could affect the levels of oxidative stress.

The most notable bioactivity of GPCP is their antioxidant properties [133]. Grape products containing polyphenols use various mechanisms such as sweeping reactive oxygen and nitrogen species [134, 135], chelating metals and minerals, modulating transcription factors, inhibiting and inducing pro-oxidant and antioxidant enzymes, and exerting synergistic effects on other antioxidants to exert their antioxidant activities [136, 137].

In fact, nuclear factor-erythroid-2-related factor-2 (Nrf2) signaling pathway, as an essential pathway for protection against oxidative stress, regulates the expression of many intracellular antioxidant genes [138]. Thus, this pathway is one of the most important therapeutic targets for the prevention and treatment of oxidative stress and its related diseases [139]. The GPCP including grape seed extract proanthocyanidin induce and activate Nrf2 [46, 138]. Grape products containing polyphenols also inhibit lipid peroxidase by increasing paraoxonase activity plasma [140], which consequently inhibits LDL oxidation [141].

Our subgroup analyses showed a significant increase in the ORAC levels in longer-term studies; this finding is confirmed by other studies [32, 44, 119]. Meta-regression analysis also indicated a significant relationship between duration of GPCP consumption and TAC, ORAC, and MDA levels. The literature indicated that supplementation duration affected the efficiency of the dietary polyphenols. Furthermore, the supplementation duration should be sufficiently long to influence the oxidative stress markers. In order to demonstrate the dose-response relationship, long-term studies are needed to exert significant effects on the antioxidant biomarkers [31, 32, 142]. The impact of grape seed extract on blood pressure was also more pronounced in longer study [83].

Our results indicated a significant elevation in SOD and ORAC levels in higher doses of grape polyphenols. The meta-regression showed that the changes in SOD, TAC, ORAC, and GPX levels were dependent on grape polyphenols supplementation dose. Similar to our results, a study indicated that different GPCP supplementation doses had different effects. For example, the flow-mediated dilation (FMD) improved better at a higher dose of red grape powder, while the diastolic blood pressure decreased better at a lower dosage of this product [123]. Contrary to our results, a meta-analysis showed that systolic and diastolic blood pressure decreased significantly at lower doses of GSE [83]. Moreover, 25 and 50% (v / v) concentrations of GPCP showed similar antioxidant activity. These concentrations were better than the 10% (v / v) concentration. These results show that the antioxidant capacity of the phenols is probably in the saturated concentration range and does not increase with higher concentrations [143].

The significant increase in SOD levels in studies with cross over designs might be due to the fact that these trials (range: 0.4–300 g, mean: 150.22 g) had significantly higher GPCP doses than the parallel trials (range: 0.1–0.35 g, mean: 0.8 g).

Subgroup analysis also indicated a significant effect of GPCP on TAC, SOD, and ORAC in healthy individuals. Similar to our results, other studies showed that the antioxidant capacity of the studied population may vary with their health status [1]. A meta-analysis found that the effect of grape seed extract on blood pressure depended on the individuals’ initial blood pressure level, although the confounding effect of the patient’s medications should be considered in unhealthy individuals [83]. Another meta-analysis reported that the elevated levels of FMD were different between the individuals with cardiovascular risk factors and the healthy participants [56]. In addition, the dose-response mechanism of the grape products may vary based on the individuals’ health status [144]. According to a previous study, supplementation with GPCP had different effects on smokers since they need higher levels of antioxidants; consequently higher doses of GPCP are required for clearer results [43]. In health status, a balance exists between production of free radicals and the antioxidant defense system that prevents the disease. In disease conditions, the balance shifts towards producing free radicals and increasing oxidative stress [145]; consequently, unhealthy people are expected to have higher levels of oxidative stress [1] and require higher doses of GPCP to improve antioxidant macros.

The present research has some strength. This is the first study over the effect of GPCP on oxidative stress. Subgroup analyses were also conducted on the study type, duration, and quality, the products’ dosage, and the participants’ health status. However, this meta-analysis had several limitations. Few oxidative biomarkers evaluated in most RCTs also, oxidative markers had rapid mechanism of oxidation, future studies should evaluate all of related biomarkers especially TAC, therefore, the results will be more accurate. The RCTs included in the present study had limited follow-up periods. Moreover, the investigated articles were heterogeneous considering their populations’ characteristics as well as the administered type and doses of GPCP. So, further clinical trials are needed over the effect of grape polyphenol on the oxidative biomarkers as primary outcome using different doses and type of GPCP. Polyphenol contents in grape products are varied widely because many factors influence their contents, such as grape cultivars, season, processing, storage condition, and duration. Future researchers are suggested to report the amount of grape polyphenol in their test products and serum levels of polyphenols in participants. Since most studies did not consider the effects of confounders, including lifestyle, diet, physical activity, smoking, health/disease, age and medications, we were unable to evaluate these effects.

## Conclusions

In conclusion, the results of this study demonstrated that supplementation with GPCP had a significant effect on increasing the TAC levels, but it had no effect on other oxidative stress biomarkers. The effect of GPCP on SOD, GPX, ORAC, and TAC levels depended on the administered dosage. In the same regard, the supplementation duration affected MDA, TAC, and ORAC levels. However, further well-designed RCTs with larger sample sizes and longer-durations are required in this area.

## Supplementary Information

**Additional file 1.**

**Additional file 2.**

**Additional file 3.**

**Additional file 4.**

**Additional file 5.**

**Additional file 6.**

**Additional file 7.**

**Additional file 8.**

**Additional file 9.**

**Additional file 10.**

**Additional file 11.**

**Additional file 12.**

**Additional file 13.**

**Additional file 14.**

**Additional file 15.**

## Data Availability

All data generated or analyzed during this study are included in this published article.

## Abbreviations

FMD: Flow-mediated dilation
GPCP: Grape Products Containing Polyphenols
GPX: Glutathione Peroxidase
GSE: Grape Seed Extract
GSH: Reduced Glutathione
MDA: Malondialdehyde
ORAC: Oxygen Radical Absorbance Capacity
PRISMA: Preferred Reporting Items for Systematic Reviews and Meta-Analysis guidelines
RCT: Randomized Clinical Trial
SOD: Superoxide Dismutase
TAC: Total Antioxidant Capacity
USA: United States of America
WMD: Weighted Mean Difference
