# Supplementary material for: The effect of grape products containing polyphenols on oxidative stress: a systematic review and meta-analysis of randomized clinical trials
Source: Nutr J. 2021 Mar 12;20:25. doi: 10.1186/s12937-021-00686-5 (PMC7971097; doi:10.1186/s12937-021-00686-5)
Supplement: Supplementary file 1 — Additional file 1. [file 12937_2021_686_MOESM1_ESM.docx]

**
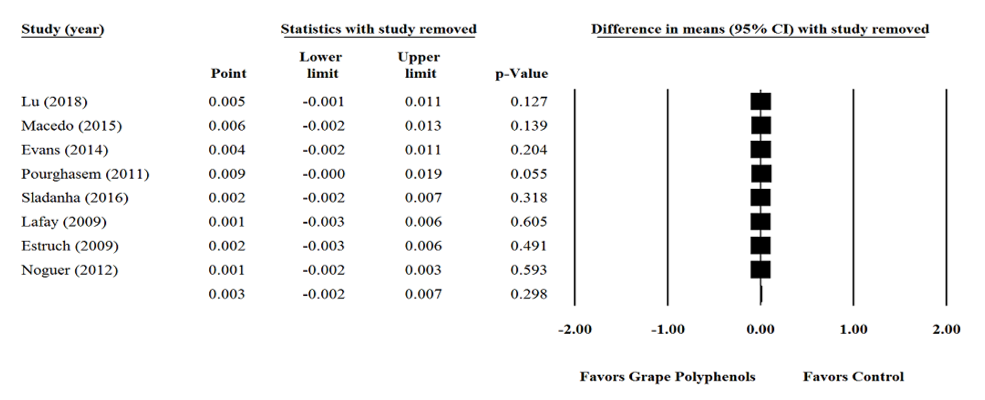
**

**Supplementary figure 1.** Sensitivity analysis was performed using a random effect model for impact of grape products containing polyphenols (GPCP) and superoxide dismutase.
