# Supplementary material for: The effect of grape products containing polyphenols on oxidative stress: a systematic review and meta-analysis of randomized clinical trials
Source: Nutr J. 2021 Mar 12;20:25. doi: 10.1186/s12937-021-00686-5 (PMC7971097; doi:10.1186/s12937-021-00686-5)
Supplement: Supplementary file 5 — Additional file 5. [file 12937_2021_686_MOESM5_ESM.docx]

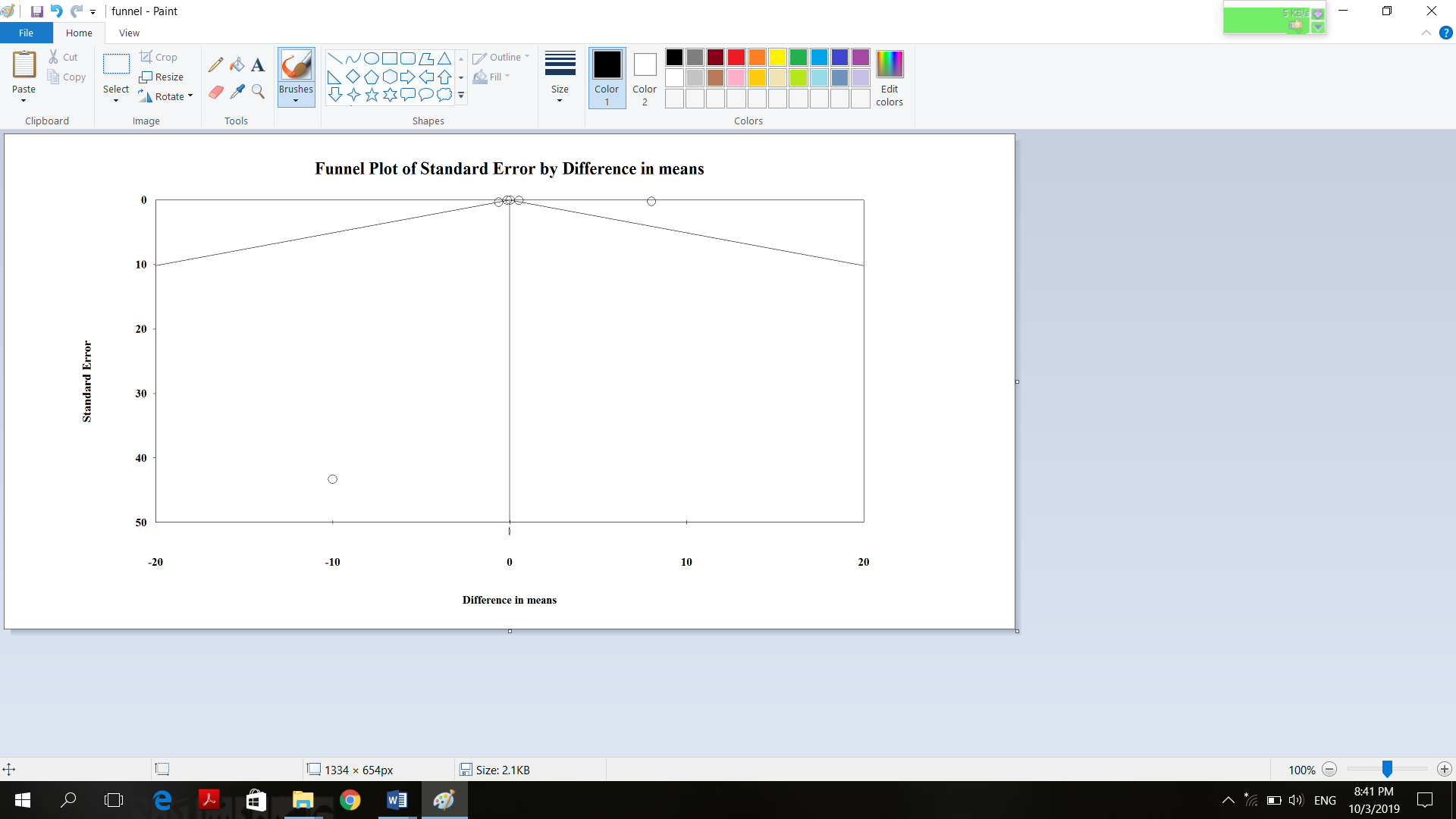


**Supplementary figure 5.** Funnel plot of weighted mean differences total antioxidant capacity concentration with grape products containing polyphenols (GPCP).
