# Supplementary material for: The effect of grape products containing polyphenols on oxidative stress: a systematic review and meta-analysis of randomized clinical trials
Source: Nutr J. 2021 Mar 12;20:25. doi: 10.1186/s12937-021-00686-5 (PMC7971097; doi:10.1186/s12937-021-00686-5)
Supplement: Supplementary file 7 — Additional file 7. [file 12937_2021_686_MOESM7_ESM.docx]

**
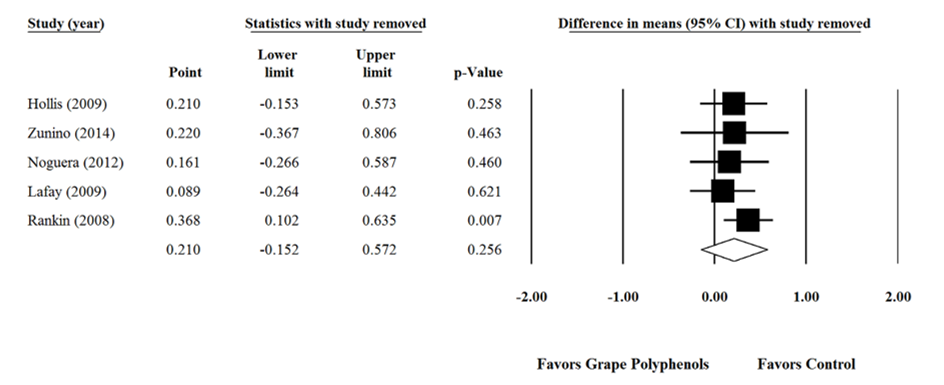
**

**Supplementary figure 7.** Sensitivity analysis was performed using a random effect model for impact of grape products containing polyphenols (GPCP) and oxygen radical absorbance capacity levels.
