# Supplementary material for: The effect of grape products containing polyphenols on oxidative stress: a systematic review and meta-analysis of randomized clinical trials
Source: Nutr J. 2021 Mar 12;20:25. doi: 10.1186/s12937-021-00686-5 (PMC7971097; doi:10.1186/s12937-021-00686-5)
Supplement: Supplementary file 9 — Additional file 9. [file 12937_2021_686_MOESM9_ESM.docx]

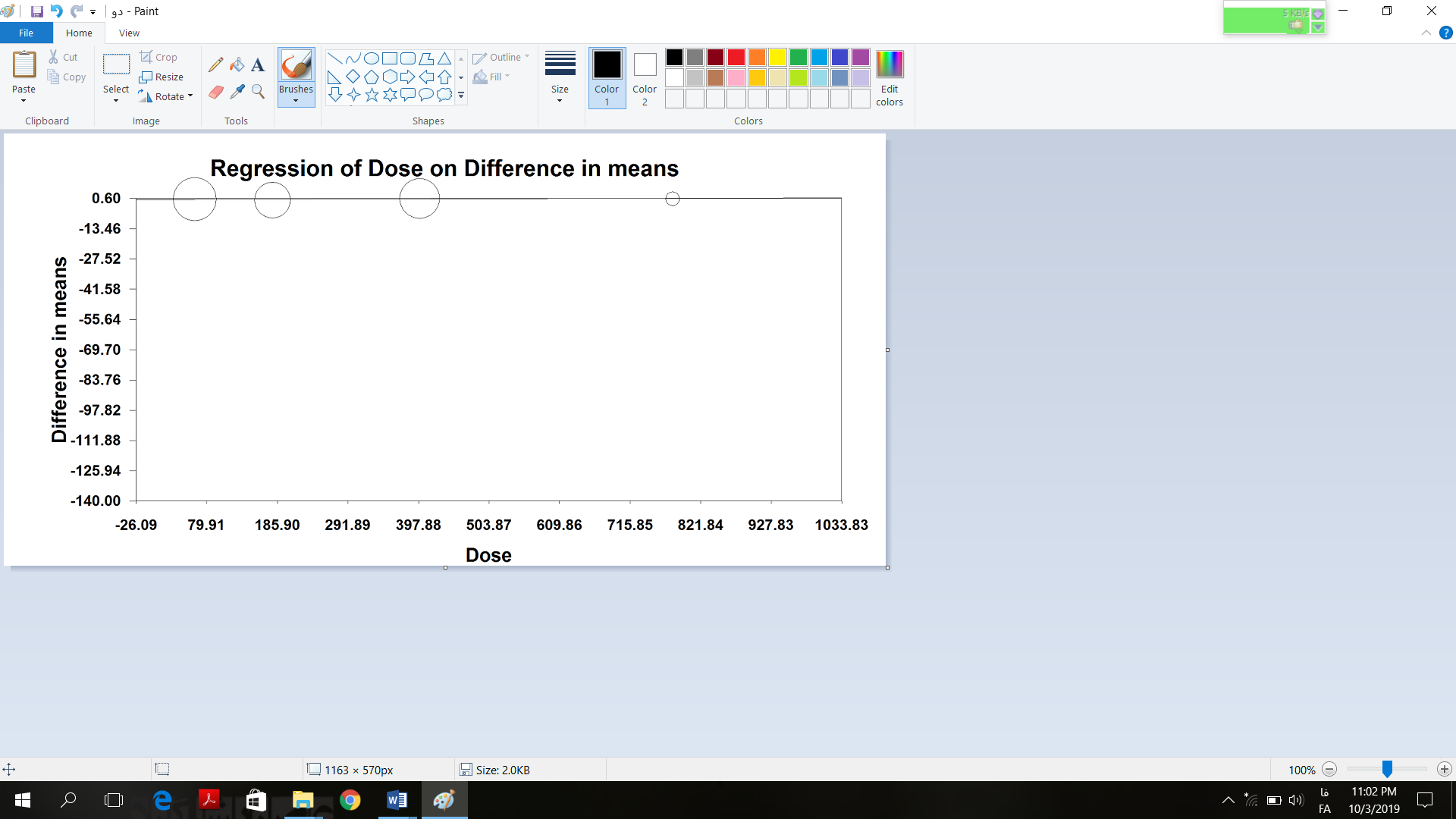


**A**


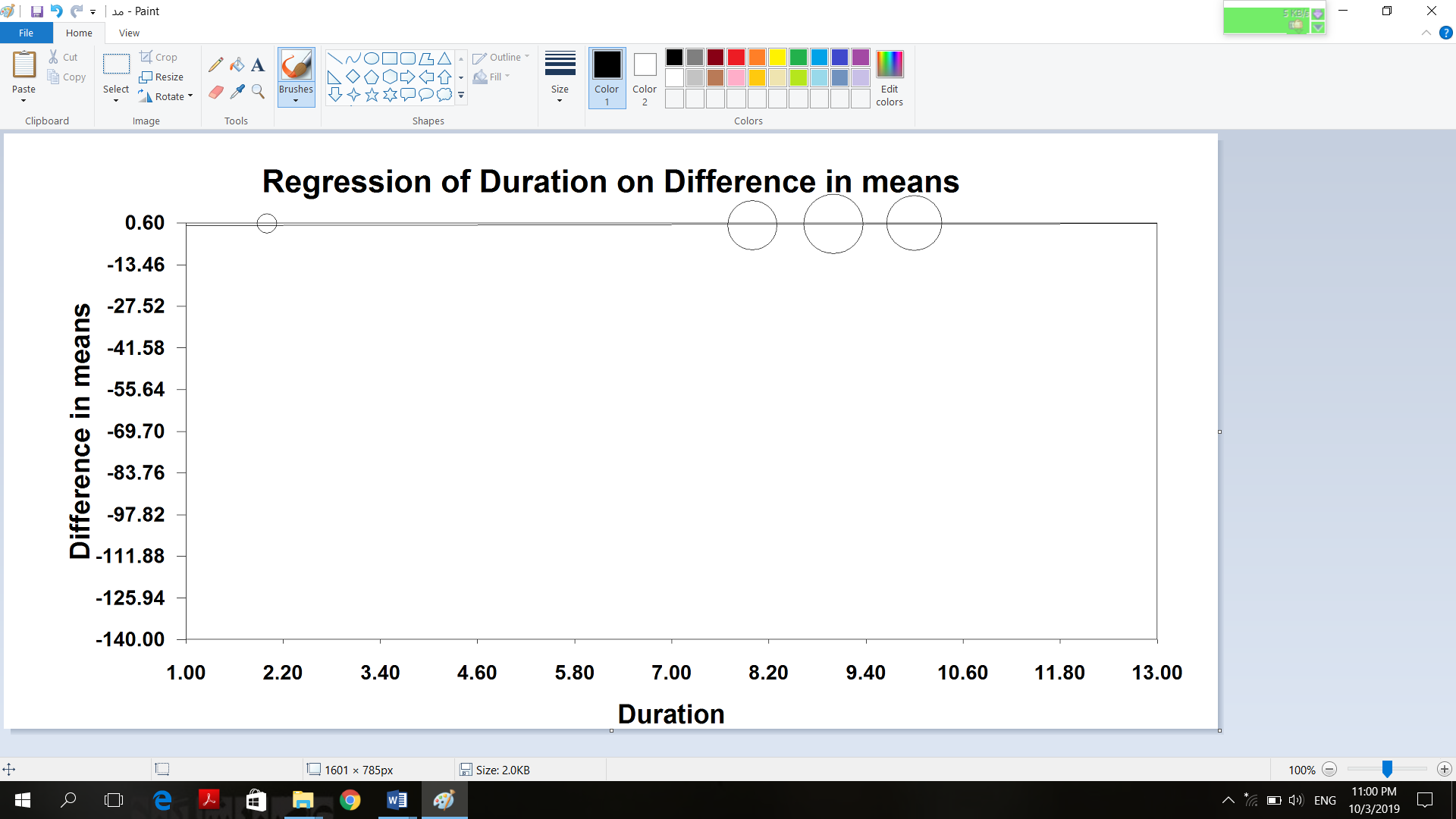


**B**

**Supplementary figure 9.** Meta-regression plots of the association between weighted mean difference in plasma oxygen radical absorbance capacity concentrations values after grape products containing polyphenols (GPCP) intake with dose (A) and duration (B) of intake. The size of each circle is inversely proportional to the variance of change.
