# Supplementary material for: The effect of grape products containing polyphenols on oxidative stress: a systematic review and meta-analysis of randomized clinical trials
Source: Nutr J. 2021 Mar 12;20:25. doi: 10.1186/s12937-021-00686-5 (PMC7971097; doi:10.1186/s12937-021-00686-5)
Supplement: Supplementary file 10 — Additional file 10. [file 12937_2021_686_MOESM10_ESM.docx]

**
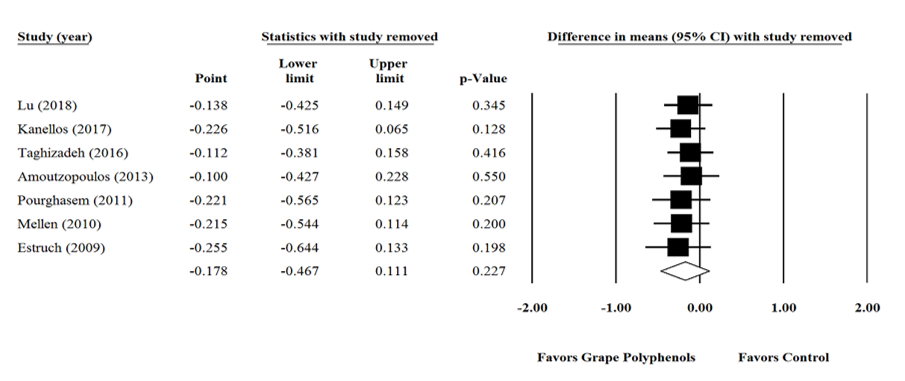
**

**Supplementary figure 10.** Sensitivity analysis was performed using a random effect model for impact of grape products containing polyphenols (GPCP) and Malondialdehyde.
