# Supplementary material for: The effect of grape products containing polyphenols on oxidative stress: a systematic review and meta-analysis of randomized clinical trials
Source: Nutr J. 2021 Mar 12;20:25. doi: 10.1186/s12937-021-00686-5 (PMC7971097; doi:10.1186/s12937-021-00686-5)
Supplement: Supplementary file 12 — Additional file 12. [file 12937_2021_686_MOESM12_ESM.docx]

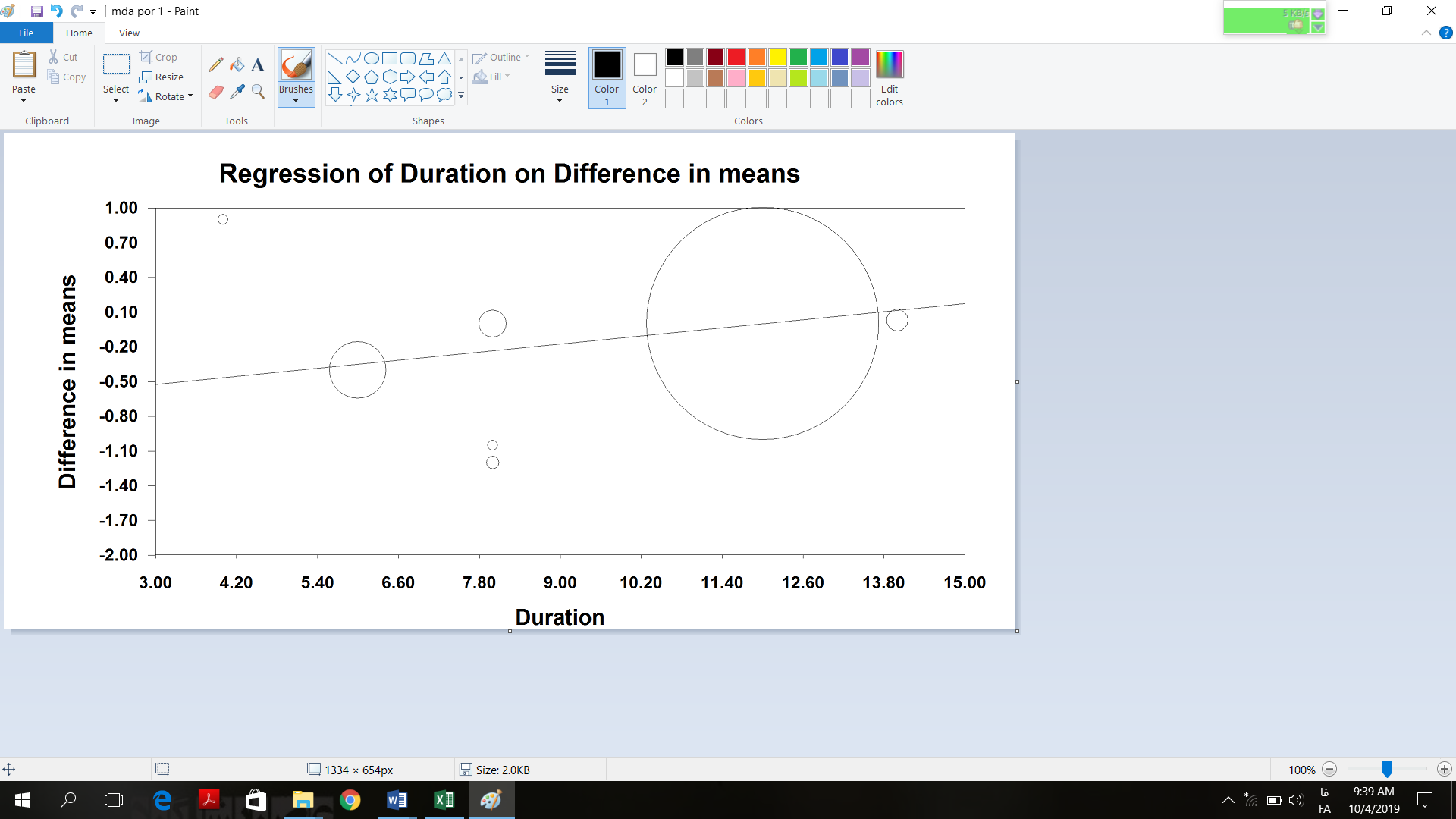


**A**


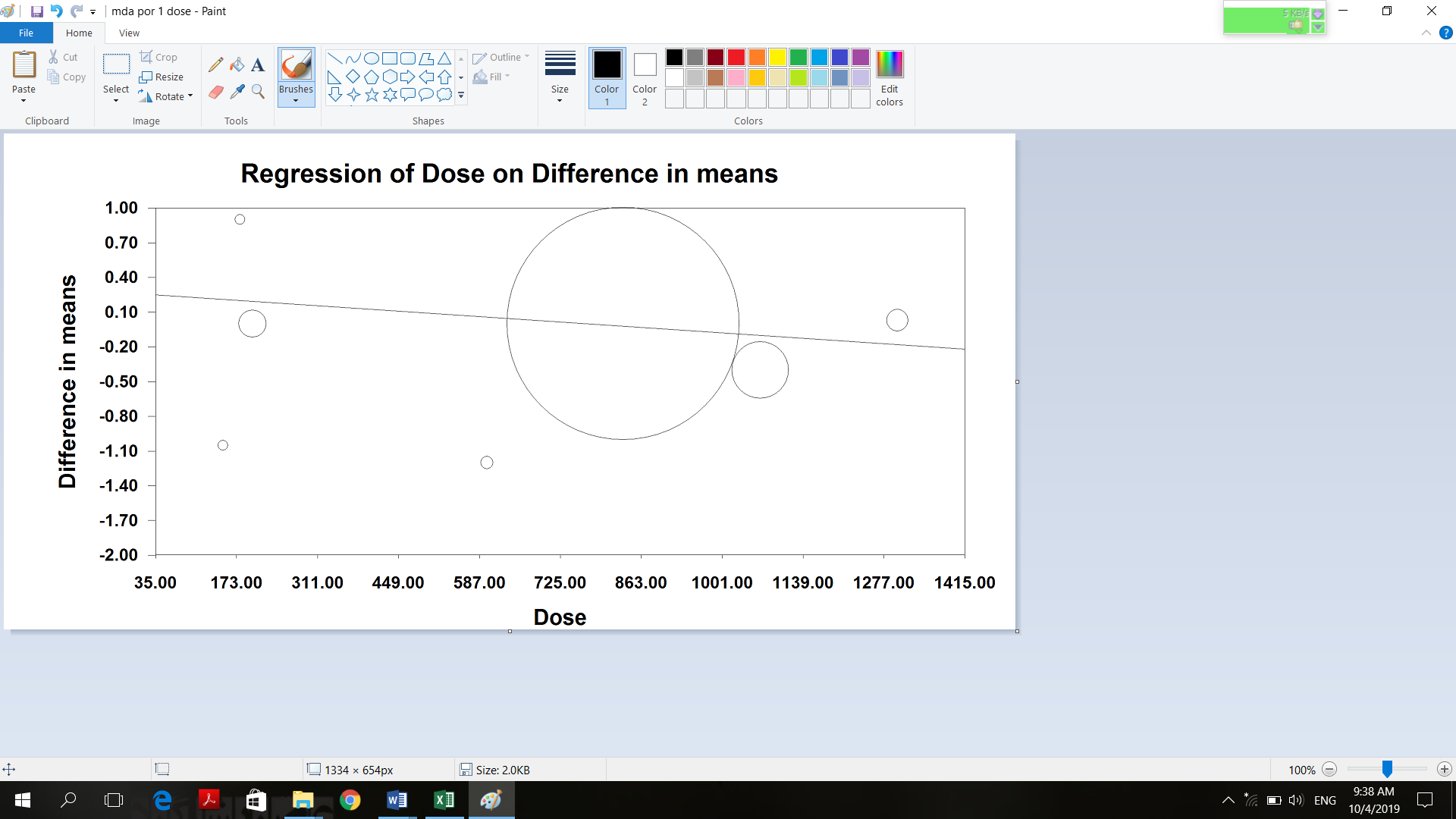


**B**

**Supplementary figure 12.** Meta-regression plots of the association between Standardized mean difference in plasma Malondialdehyde concentrations values after grape products containing polyphenols (GPCP) intake with duration (A) and dose (B) of intake. The size of each circle is inversely proportional to the variance of change.
