# Supplementary material for: The effect of grape products containing polyphenols on oxidative stress: a systematic review and meta-analysis of randomized clinical trials
Source: Nutr J. 2021 Mar 12;20:25. doi: 10.1186/s12937-021-00686-5 (PMC7971097; doi:10.1186/s12937-021-00686-5)
Supplement: Supplementary file 15 — Additional file 15. [file 12937_2021_686_MOESM15_ESM.docx]

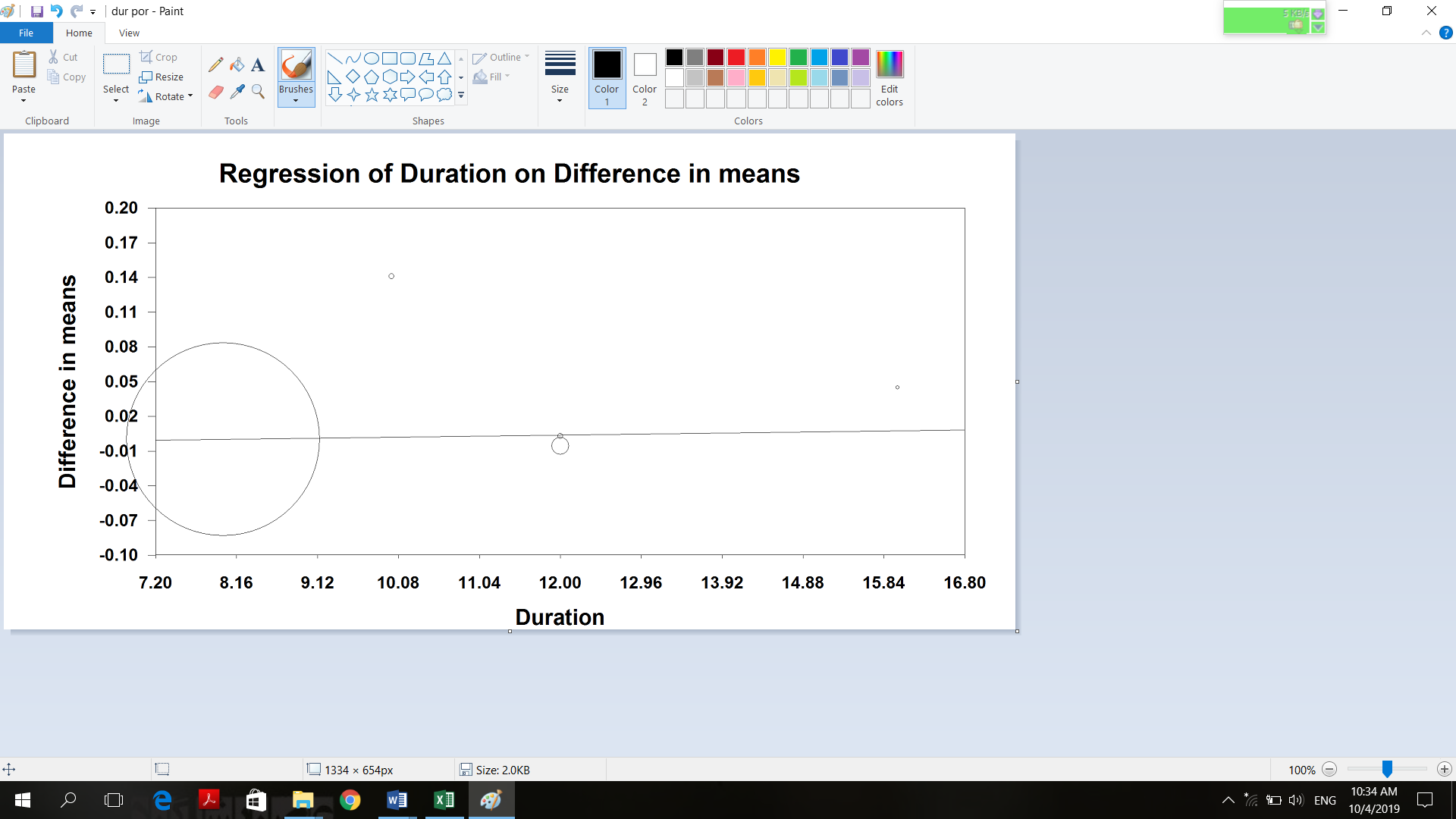


**A**


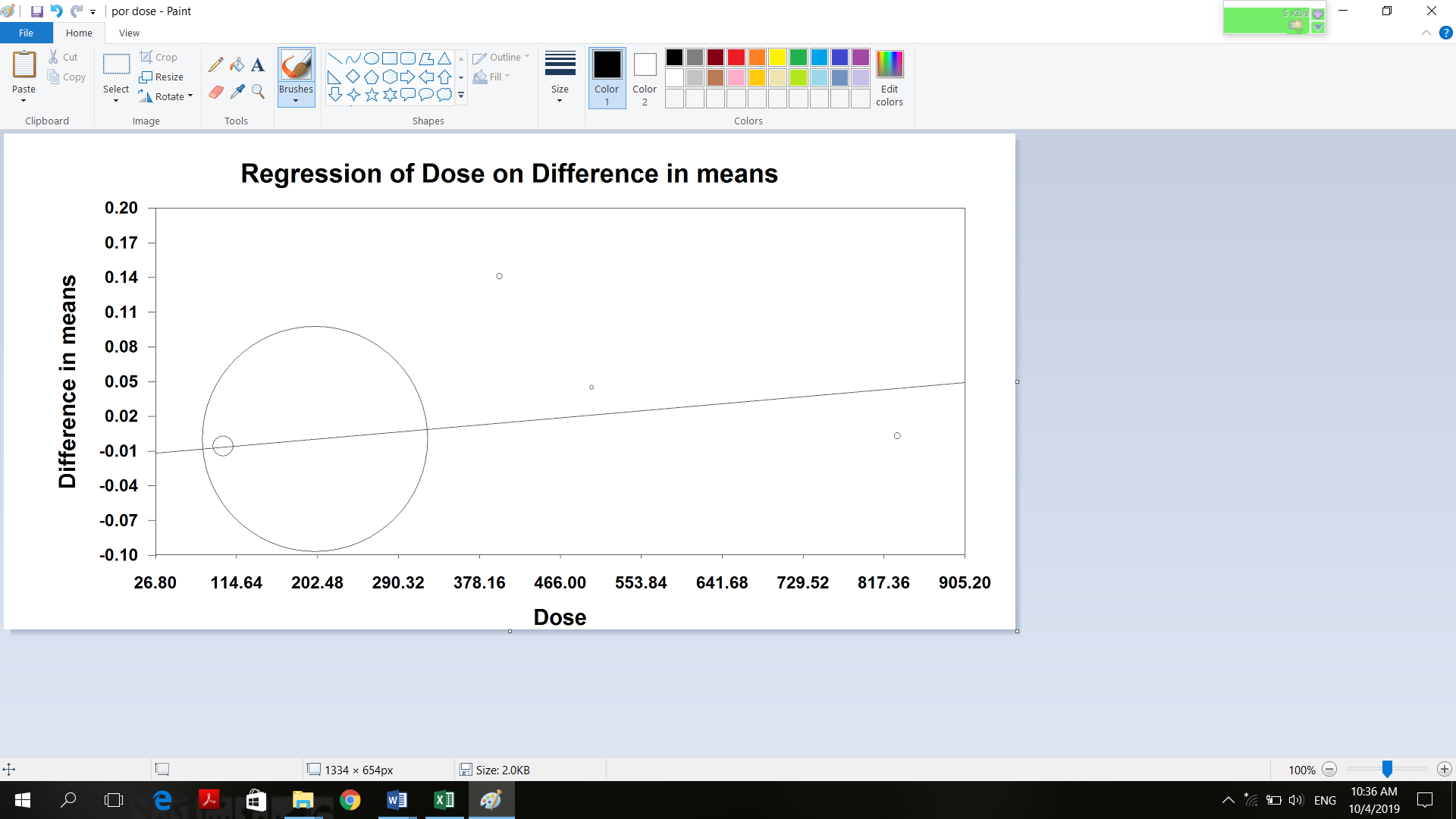


**B**

**Supplementary Figure 13.** Meta-regression plots of the association between Standardized mean difference in plasma glutathione peroxidase concentrations values after grape products containing polyphenols (GPCP) intake with duration (A) and dose (B) of intake. The size of each circle is inversely proportional to the variance of change.
